# Efficient Generation of Fully Reprogrammed Human iPS Cells via Polycistronic Retroviral Vector and a New Cocktail of Chemical Compounds

**DOI:** 10.1371/journal.pone.0026592

**Published:** 2011-10-26

**Authors:** Zhonghui Zhang, Yongxing Gao, Albert Gordon, Zack Z. Wang, Zhijian Qian, Wen-Shu Wu

**Affiliations:** 1 Stem and Progenitor Cell Biology Program, Center for Molecular Medicine, Maine Medical Center Research Institute, Maine Medical Center, Scarborough, Maine, United States of America; 2 Section of Hematology/Oncology, Department of Medicine, University of Illinois at Chicago, Chicago, Illinois, United States of America; 3 Children's Hospital Oakland Research Institute, Oakland, California, United States of America; University of Nebraska Medical Center, United States of America

## Abstract

Direct reprogramming of human somatic cells into induced pluripotent stem (iPS) cells by defined transcription factors (TFs) provides great potential for regenerative medicine and biomedical research. This procedure has many challenges, including low reprogramming efficiency, many partially reprogrammed colonies, somatic coding mutations in the genome, etc. Here, we describe a simple approach for generating fully reprogrammed human iPS cells by using a single polycistronic retroviral vector expressing four human TFs in a single open reading frame (ORF), combined with a cocktail containing three small molecules (Sodium butyrate, SB431542, and PD0325901). Our results demonstrate that human iPS cells generated by this approach express human ES cells markers and exhibit pluripotency demonstrated by their abilities to differentiate into the three germ layers *in vitro* and *in vivo*. Notably, this approach not only provides a much faster reprogramming process but also significantly diminishes partially reprogrammed iPS cell colonies, thus facilitating efficient isolation of desired fully reprogrammed iPS cell colonies.

## Introduction

Human embryonic stem (ES) cells have great therapeutic potential for treatment of various diseases, but the generation of these cells is ethically controversial. Therefore, generation of pluripotent stem cells from somatic cells holds promise for regenerative medicine and biomedical research [1]. Reprogramming of somatic cells to pluripotent ES cell-like cells, termed induced pluripotent stem (iPS) cells, has been achieved by the expression of defined transcription factors (TFs), including either the combination of Oct4, Klf4, Sox2 and c-Myc, or Oct4, Sox2, Nanog, and Lin28 [2], [3], [4], [5], [6]. This method facilitates the generation of patient- and disease-specific pluripotent stem cells without immune rejection and ethical controversy that are valuable for clinical applications.

Reprogramming human somatic cells by integrating approaches (i.e. lentiviral or retroviral infection of defined TFs) is very inefficient (up to 0.05%) with slow kinetics (almost 4 weeks), and requires very high transduction efficiency [7]. Although developed non-integrating approaches generate human iPS cells, such as adenovirus-mediated gene delivery [8], non-integrative episomal vectors [9], [10], piggyBac transposition [11], self-excisable vector [12], mRNAs [13], non-viral minicircle vector [14], or by the delivery of reprogramming protein [15], the same issue is evident in reprogramming for of iPS cell applications. Compared with non-integrating approaches, current integrating approaches for generating human iPS cells have much higher efficiency, and are therefore efficacious approaches for generating human iPS cells.

Recently, rapid progress has been made towards enhancing the efficiency of human iPSC generation by using integrating approaches in combination with small molecules. Butyrate, a small-chain fatty acid histone deacetylase inhibitor, greatly enhances the efficiency of generating murine and human iPS cells with TFs in a single or four individual vectors [16], [17]. Inhibition of both TGFβ and MAPK/ERK pathways using small molecules (SB413542 and PD0325901, respectively) enhances reprogramming of human fibroblasts with the four individual TFs [18]. Interestingly, a combination of A-83-01 and PD0325901 generates iPS cells from neonatal human epidermal keratinocytes by only two TFs [19]. Although these small molecules increase efficiency of generating human iPS cells, they also require transduction of individual TFs. Thus, there is still a tremendous need for a safer more efficient approach for generating fully reprogrammed human iPS cells.

Here, we describe a simple approach for generating fully reprogrammed human iPS cells by using a single vector expressing four TFs in a single open reading frame (ORF), in combination with a cocktail containing three small molecules.

Our results demonstrate that human iPS cells generated by this approach express human ES cell markers and exhibit pluripotency, as demonstrated by their abilities to differentiate into the three germ layers in vitro and in vivo. Notably, the reprogramming process is much faster (within two weeks), the percentage of fully reprogrammed human iPS cell colonies generated by this approach is significantly high, and most of iPS cells generated by our approach contain a single copy of polycistronic retroviral vector. We provide a new alternative approach to generate full reprogrammed human iPS cells with high efficiency.

## Results

### A polycistronic retroviral vector for co-expression of four defined TFs

**Figure 1:**
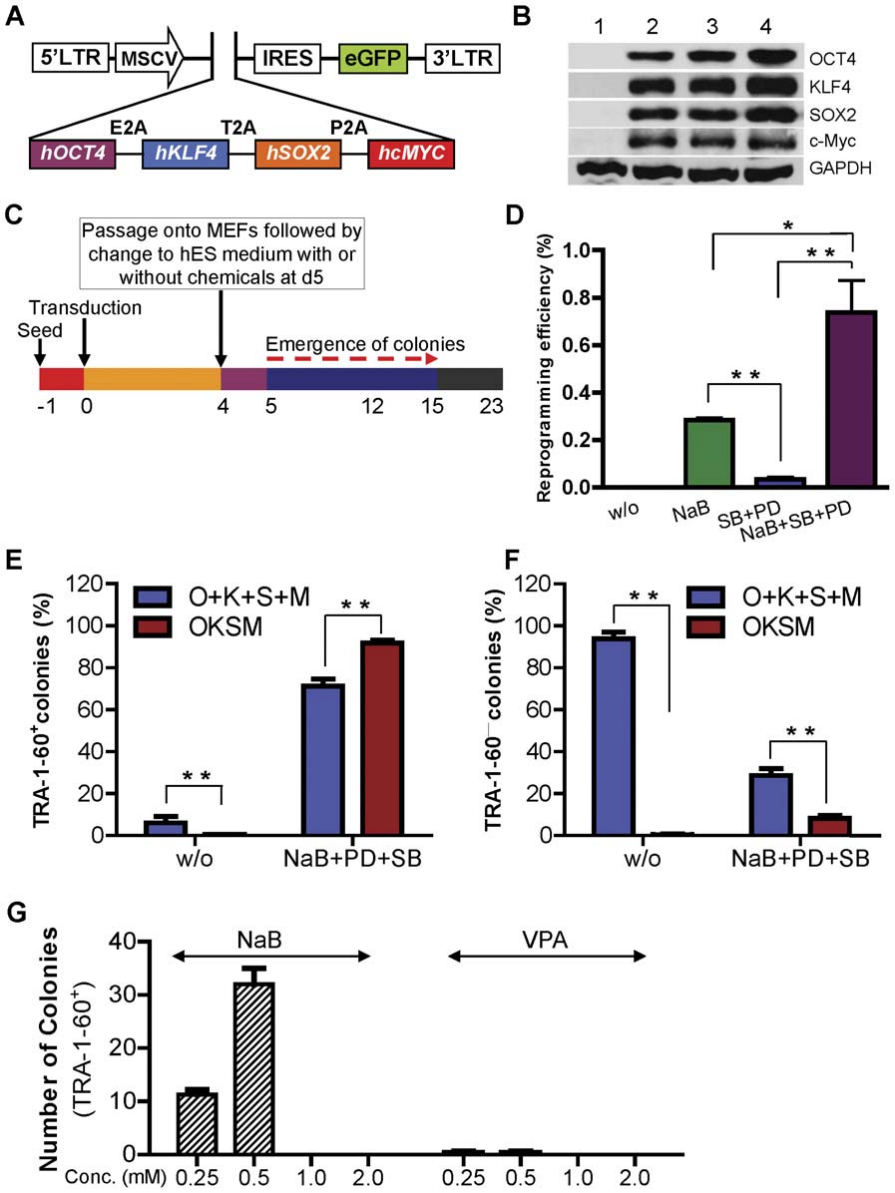
Reprogramming of human adult by using a single polycistronic retroviral vector expressing four TFs combined in small molecules. (**A**) Schematic representation of retroviral expression vector (pMIGR1) expressing four TFs linked via 2A sequences. Four defined human TFs (OCT3/4, KLF4, SOX2, and c-MYC) were fused in-frame via 2A sequences and coexpressed as a single ORF (designated *OKSM*). The 2A-linked cassette and GFP marker, which were separated by an internal ribosome entry site (IRES) sequence, were driven by an MSCV promoter. (**B**) Western-blot analysis of four TFs from the *hOKSM* fusion gene. pMIGR1-*hOKSM* (lane 2 and 3, two clones) was transiently transfected into U2OS cells, and expression of each TF was confirmed by immunoblotting with each corresponding antibodies. Expression plasmids (lane 4) expressing OCT3/4, KLF4, SOX2, or c-MYC cDNA were included as positive controls; empty vector (pMIGR1, lane 1) was included as a negative control. (**C**) A schematic diagram of the reprogramming protocol used. Human fibroblasts were plated at day-1 and transduced by retroviral polycistronic vector at day-0. The transduced cells were cultured for 4 days in the FBS-containing medium used for fibroblasts, and then were harvested and counted. The single-cell suspension was plated onto a preformed MEF monolayer. The cell media were changed the next day to hES medium with or without small molecules. Concentrations of the small molecules used in these experiments were: NaB (sodium butyrate): 0.5 mM; SB (SB431542): 2 µM; PD (PD0325901): 0.5 µM. Emergence of colonies was monitored daily until discernible hES-like colonies were picked (days 12–23). (**D**) The reprogramming efficiency by pMIGR1-*hOKSM* using the modified protocol with various combinations of small molecules as indicated. (**E**) The percentage of TRA-1-60^+^ iPS cell colonies among total colonies generated by *OKSM* or four individual TFs (O, Oct4; K, Klf4; S, Sox2; M, c-Myc). Human fibroblasts were infected with pMIGR1-*hOKSM* or a mixture of retroviruses expressing four individual TFs, seeded on feeder cells, and then treated with the chemical compounds (NaB, SB, and PD) as described in (C) or left without treatment. (**F**) The percentage of partially reprogrammed iPS cell colonies (TRA-1-60^−^). (**G**) Total number of iPS cell colonies (TRA-1-60^+^) generated by *OKSM* in combination with NaB or VPA. Human fibroblasts infected with pMIGR1-*hOKSM* retroviruses were seeded on feeder cells and then treated with a increasing concentration (0.25, 0.5, 1.0, and 2.0 mM) of NaB or VPA. Cell colonies were stained with TRA-1-60-specific antibody. For all figures, the Mean and SD values were from at least two duplicates and *p<0.05 and **p<0.01.

We previously reported generation of murine iPSC from MEFs by using our polycistronic lentiviral vector containing the murine *KOSM* fusion gene [20]. Most murine iPS colonies generated by our system contain only one copy of viral vector leading to a decrease of multiple proviral copies throughout the genome. When transduced into human fibroblast cells, we found that this system generated very few ES-like cell colonies (data not shown). We reasoned that human TFs may increase reprogramming efficiency in human somatic cells. Thus, we constructed a human *OKSM* fusion gene with self-cleaving 2A sequences into a retroviral vector (Figure 1A). A humanized GFP marker was cloned downstream of the *OKSM* gene that was separated by an IRES to enable tracking of transgene expression during the reprogramming process. The self-cleaving 2A sequences derived from the foot - and – mouth disease viruses are small and can efficiently cleave polycistrons at specific site [21]. We and others used 2A sequences to co-express defined TFs for generation of iPS cells [20], [22], [23], [24].

We transfected pMIGR1-*hOKSM* into cells to verify that the *OKSM* fusion product can be processed efficiently into individual proteins with correct size confirmed by western blot analysis, and to compare each protein translated from each expression vector (Figure 1B).

### Chemical compounds greatly enhance human iPSC generation by using a single polycistronic vector

Next, we prepared retroviruses from this vector and infected them into human fibroblasts (Figure 1C). As expected, retroviruses carrying the *hOKSM* gene were efficiently transduced into human fibroblasts, and the EGFP marker was clearly visualized by fluorescent microscopy (Figure 1D). The fraction of infected cells (GFP+) was 85%, as determined by flow cytometry (data not shown). Infected and uninfected fibroblasts were harvested four days after transduction, resuspended as a single cells, plated on MEF feeders, and cultured as hES cells with standard hES medium (Figure 1C). In ten individual experiments, we did not obtain any hES cell-like colonies. Thus, this vector alone did not efficiently generate iPS cells from human fibroblasts in conventional culture medium.

Recently, it was shown that TFs-driven reprogramming of somatic cells could be significantly enhanced by small molecules, such as sodium butyrate [16], [17], SB431542, and PD0325901 [18]. Sodium butyrate is an inhibitor of histone deacetylases, and it increased reprogramming efficiency of both mouse and human somatic cells [16], [17]. SB431542 is a potent and selective inhibitor of the transforming growth factor-β (TGF-β) type I receptor activin receptor-like kinase ALK5 (IC_50_ = 94 nM), and its relatives: ALK4 and ALK7 [25], [26]. PD0325901 is a MEK inhibitor [27]. Ding and his coworkers showed that the reprogramming efficiency of human primary fibroblasts was increased after infection with a mixture of retroviruses expressing each TFs in combination with a cocktail consisting of SB431542, PD0325901, and thiazovivin [18]. Therefore, we decided to determine if the addition of three compounds (butyrate, SB431542, and PD0325901) will promote a reprogramming process in primary human fibroblasts infected with pMIGR1-*hOKSM* (Figure 1C). We found that hES cell-like colonies emerged from 0.29% of infected cells 15 days after viral infection by adding butyrate alone (called one-molecule treatment) (Figure 1D).

To study these colonies in more detail, we picked up 23 colonies based on hES cell morphology between 15 to 23 days after infection, and expanded them for further analysis. We first performed alkaline phosphatase (AP) staining and then stained these colonies with the reliable Tra-1-60 specific antibody to monitore complete reprogramming of human somatic cells [28]. The results showed that 100% of AP-positive colonies were positive for Tra-1-60 marker (data not shown). We also tested a two molecule cocktail (SB431542 and PD0325901, SB+PD) and a three molecule cocktail (sodium butyrate together with SB431542 and PD0325901, NaB+SB+PD) for reprogramming. Notably, treatment with the three molecule cocktail (NaB+SB+PD) significantly increased reprogramming efficiency (∼3-fold) and kinetics (12 days after infection), when compared the groups treated with sodium butyrate alone. By using this approach, we achieved as high as a ∼0.93% reprogramming efficiency based on the number of AP^+^ colonies formed from *hOKSM*-infected human fibroblasts (Figure 1C, 1D). The two-molecule treatment was less potent than sodium butyrate, but also increased reprogramming efficiency (∼0.05%), compared to the untreated control.

In addition, we evaluated if our system would readily reprogram other human somatic cell types: human foreskin fibroblasts (HFF-1) and human hepatocytes. The results showed ∼0.76% and ∼2.38% reprogramming efficiency based on the number of AP^+^ colonies formed from *hOKSM*-infected human hepatocytes and HFF-1, respectively (Supplementary Table S1).

### Small molecules cocktail selectively promote generation of fully reprogrammed iPS cell colonies from human fibroblasts transduced with retroviruses expressing *hOKSM*

Because the generation of human iPS cells was dramatically enhanced by the three molecule cocktail (Figure 1D), we asked whether or not the small molecule cocktail could selectively augment *hOKSM* to fully reprogram human fibroblasts. To address this question, we infected human fibroblasts with retroviruses expressing *hOKSM* or 4 individual human TFs (Oct4, Klf4, Sox2, c-Myc) and treated with the three molecule cocktail at 4 days after being seeded on feeder cells, as shown in (Figure 1C). We stained the iPS cell colonies with the fluorescence-labeled TRA-1-60 antibody. Figure 1E shows that although without small molecules the percentage of TRA-1-60^+^ iPS cell colonies is significantly higher when cells were infected with retroviruses expressing 4 individual TFs, *hOKSM*-expressing retroviruses generated a significantly higher number of TRA-1-60^+^ iPS cell colonies than 4 individual TFs. Notably, the percentage of partially reprogrammed iPS cell colonies (TRA-1-60^−^) was 4-fold lower in the group transduced with *hOKSM* retroviruses than the group infected with 4 individual TFs (Figure 1F). Together, our data indicate that combination of *hOKSM* with the three molecule cocktail selectively promote generation of fully reprogrammed iPS cell and suppressed the growth of partially reprogrammed iPS cell colonies.

It was recently reported that NaB is much more reliable and efficient than VPA, another histone deacetylase inhibitor, for the generation of iPS cell colonies. Under *hOKSM* condition, we compared NaB and VPA on reprogramming human fibroblasts by infecting human fibroblasts with retroviruses expressing *hOKSM*, seeding these cells on feeder cells, and culturing them in hES medium containing an increasing concentration of NaB or VPA. By immunofluorescence staining, we found that the effective concentration is 0.25 and 0.5 mM for both NaB and VPA, and that NaB was 10 and 30 times more effective than VPA at each concentration, respectively, in the reprogramming of human fibroblasts into iPS cells (Figure 1G).

### Transgene silencing and integration during reprogramming and karyotype analysis

**Figure 2:**
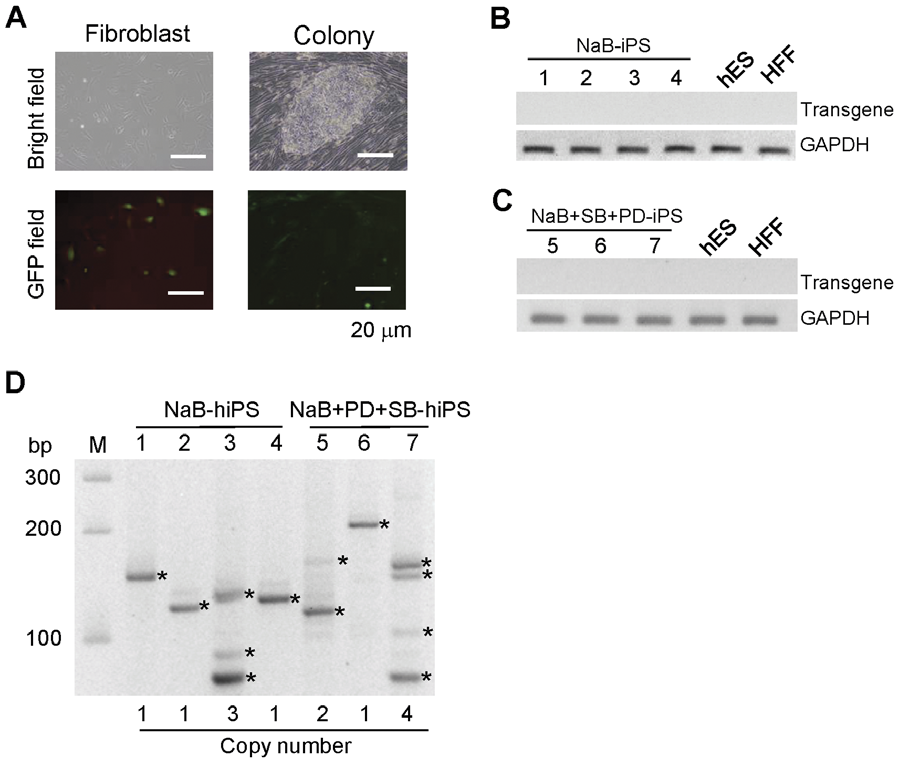
Silencing of transgene *hOKSM* during reprogramming and copy number of integrated retroviral vectors in iPS cell colonies. (**A**) Images of GFP fluorescence for tracking expression of *OKSM* transgene during reprogramming. Scale bar, 20 µm. (**B–C**) RT-PCR analysis of *hOKSM* transgene gene in iPS cell lines generated by one molecule (B) and three molecules (C). (**D**) Splinkerette-PCR analysis of retroviral integration sites in hiPS cells. Genomic DNA was extracted from each hiPS cell line and processed for splinkerette-PCR analysis with specific primers. The number of integrated retroviral vector is shown at bottom. M, 100 bp DNA marker.

The GFP marker and *hOKSM* fusion gene are driven by a common CMV promoter in pMIGR1-*hOKSM* vector. In addition, GFP expression should reveal expression of the *hOKSM* transgene. Therefore, we examined gene-silencing of the integrated transgene (*hOKSM*) during the reprogramming process by monitoring GFP marker expression. By observing GFP expression in individual iPS cell colonies, we found that GFP expression was almost undetectable at 15 days for butyrate treatment (Figure 2A). To further confirm transgene *hOKSM* silencing in these cell colonies, we examined transcripts of the *hOKSM* transgene in each iPS cell colony by RT-PCR. We synthesized cDNAs from total RNA extracted from each colony and performed PCR with a pair of specific primers for the P2A linker (Figure 1A) between the *SOX2* and *c-MYC* sequences (Supplementary information, Table S2, for primer sequences). As expected, RT-PCR analysis indicated that the transcripts of *hOKSM* transgene were not detectable in these iPS cell lines (Figures 2B, 2C).

Using a single polycistronic retroviral vector for reprogramming offers a potential advantage because fewer retroviral vectors are integrated in the genome of reprogrammed cells. Indeed, we found that iPS cells generated from MEFs using a single polycistronic retroviral vector contained only one retroviral integrating site [20]. Thus, we decided to determine the copy number of integrated retroviral vectors in the generated iPS cell colonies. By splinkerette-PCR [29], we found that 4 of the 7 iPS cell colonies contained only 1 copy number of integrated retroviral vector (Figure 2D).

**Figure 3:**
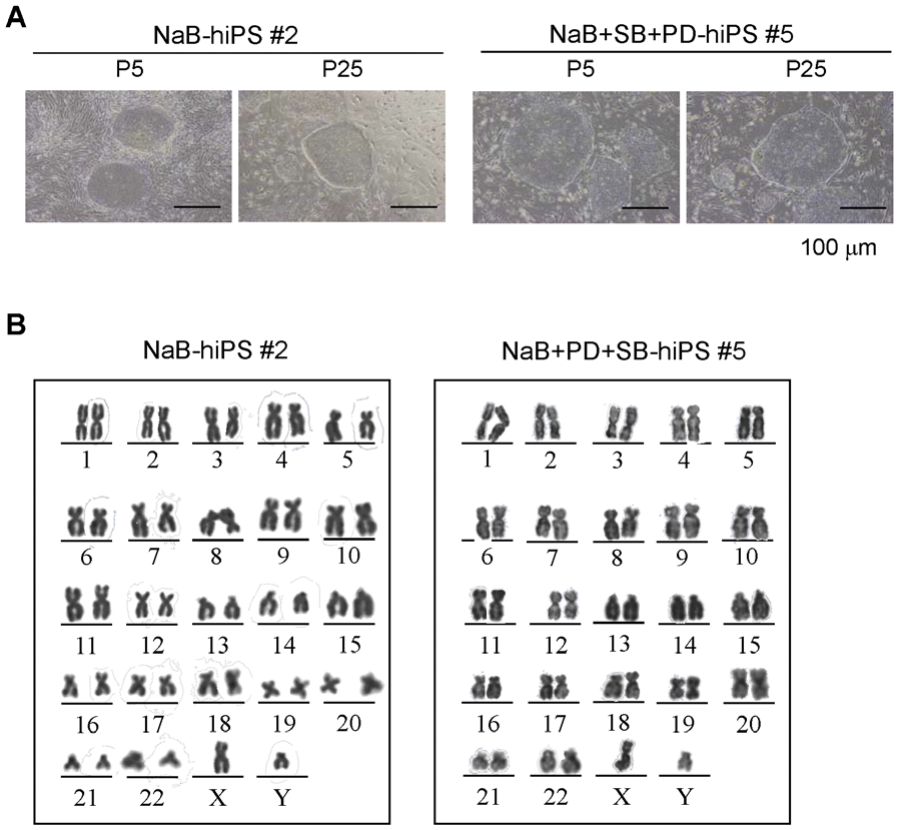
Establishment of stable hiPS cell lines generated from human fibroblasts infected with the retroviruses containing the *hOKSM* fusion gene with one or three compound treatments. (**A**) After reprogramming by combining transgene *OKSM* with small molecules, ES-like cell colonies were individually picked and expanded by serial passages under standard culture for hES cells. Scale bars, 100 µm. (**B**) G-banding karyotype analysis of iPS cell lines generated with *hOKSM* in combined with one and three small molecules. iPS cell line #2 and #5 reveal a normal karyotype.

To investigate if iPSC colonies generated using butyrate alone and the three molecule cocktail could be stably expanded under conventional hES culture conditions, we picked up four colonies from butyrate treatment and three from the three molecule cocktail treatment. All seven colonies generated in both treatments were expanded as distinct colonies with morphology similar to hES cells (Figure 3A), and they exhibited a normal karyotype after being cultured for five passages by chromosomal G-band analysis (Figure 3B).

### Pluripotency of butyrate and three molecule cocktail-treated hiPS cell lines

**Figure 4:**
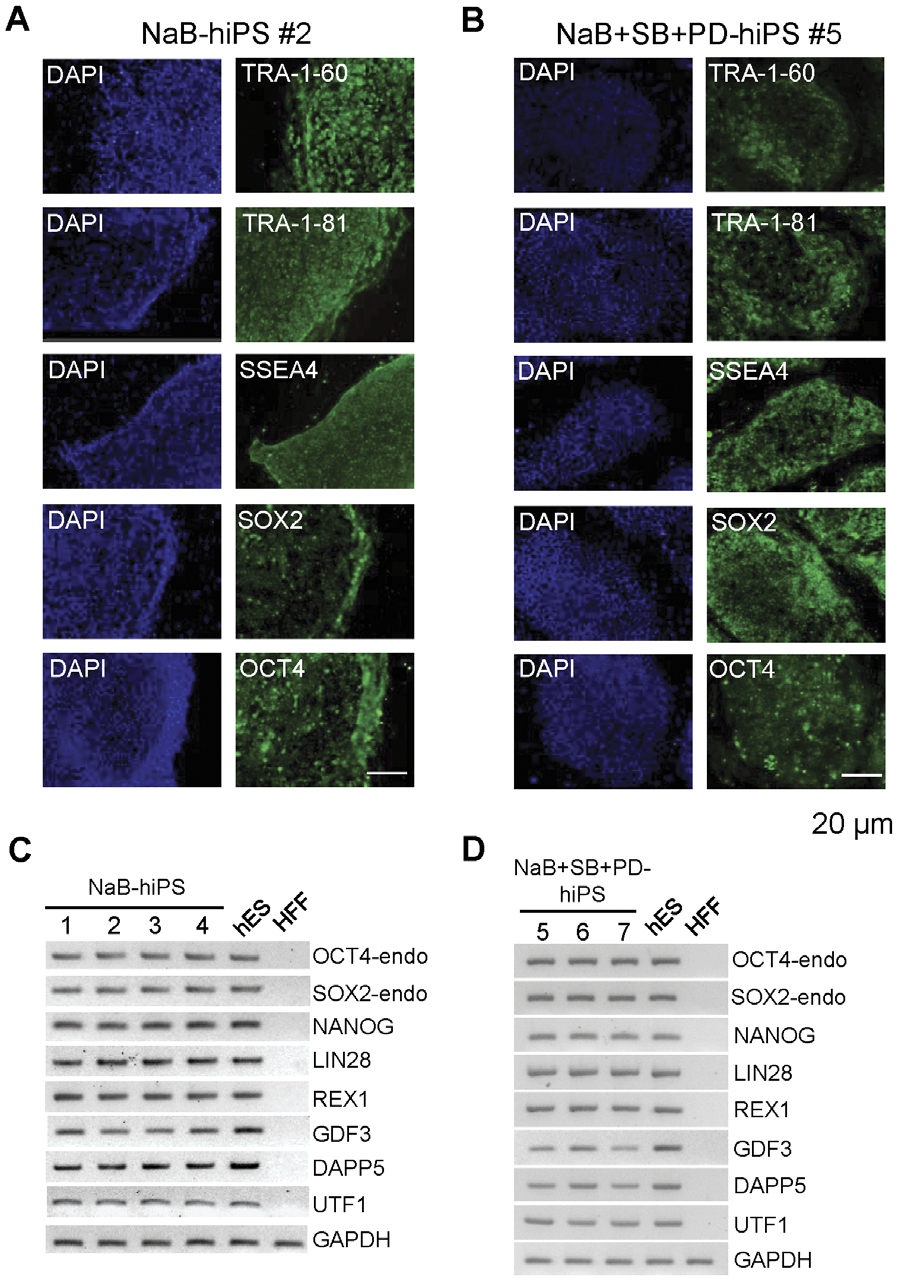
Characterization of hiPS cell lines generated from human fibroblasts using retroviruses expressing *OKSM* fusion gene and one or three small molecules. (**A, B**) Immunofluorescence staining of pluripotency markers (TRA-1-60, TRA-1-81, SSEA4, SOX2, and OCT4) in hiPS cell lines. Scale bar, 20 µm. (**C, D**) Semi-quantitative RT-PCR analysis of pluripotency markers in hiPS cell lines.

To further investigate if hiPS cell lines established by using our OKSM reprogramming system combine with butyrate or three molecule cocktail treatment, we performed immunofluorescence staining with antibodies specific for hES cell markers and semiquantative RT-PCR analysis. Immunostaining results showed that all seven colonies were positive for hES cell markers: Tra-1-60, Tra-1-81, SSEA4, OCT4, and SOX2 (Figure 4A, B). In addition, we synthesized cDNA from iPS cell lines and confirmed gene expression of multiple pluripotency markers in these cell colonies by semiquantative RT-PCR analysis. The results showed that the expression of several stem cell markers was indistinguishable from hES cells (Figure 4C, D).

**Figure 5:**
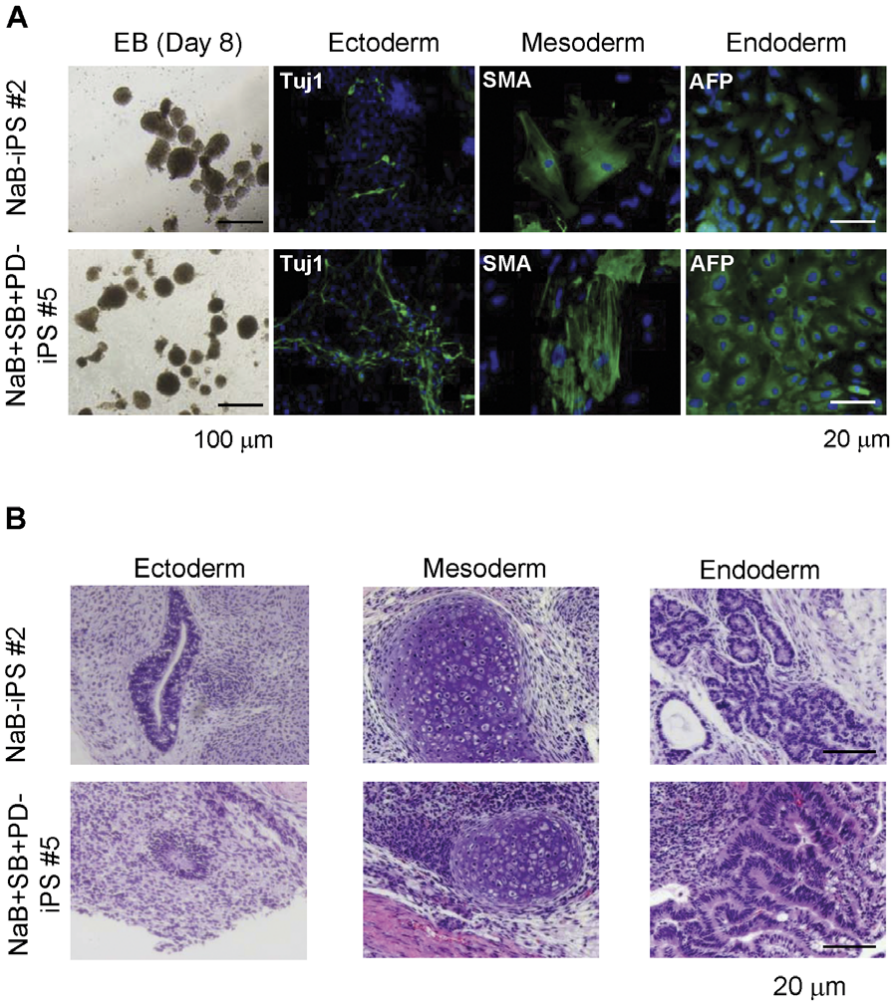
In vitro and in vivo differentiation of a single polycistronic vector-induced hiPS cell lines with one or three compounds treatment. (**A**) Micrographs of embryoid bodies (EB) generated from hiPS cel lines and in vitro differentiation. Ectodermal, mesodermal, and endodermal cell types were revealed by antibodies specific for markers βIII-Tubulin (TUJ1), SMA, and AFP, respectively. Scale bar, 100 µm (embryoid bodies) and 20 µm (all others). (**B**) In vivo differentiation assay by teratoma formation. hiPS cells (1×10^7^) were injected subcutaneously in NOD-SCID mice and teratomas were dissected after 3–8 weeks. Sections from excised tumors were stained by hematoxylin and eosin for histology analysis. Left: squamous epithelium (ectoderm); Middle: cartilage (mesoderm); Right: glandular epithelium (endoderm). Scale bar, 20 µm.

Using embryoid body (EB) formation, we further assessed the pluripotency of each hiPS cell line by differentiating them into the three embryonic gem layers in vitro. By immunostaining with anti-Tuj1 (ectoderm), anti-SMA (mesoderm), and anti-AFP (endoderm) following EB differentiation, we showed that all iPS cell colonies had the ability to generate all three embryonic germ layers (Figure 5A). Finally, we assessed the pluripotency of iPS cell lines by injecting two iPS cell lines (lines #2 and #5) into NOD-SCID mice. Our data showed that formed teratomas contain tissues derived from all three embryonic germ layers, thereby demonstrating their pluripotency (Figure 5B).

## Discussion

In the present study, we generated hiPS cells from different human somatic cells by our new reprogramming system in a small molecule cocktail when viral transduction efficiency was 25% (data not shown). Notably, the ratio of fully reprogrammed iPS colonies among AP^+^ colonies was very high (∼100%), as determined by immunostaining with anti-Tra-1-60 antibodies.

The current reprogramming approaches for human iPS cells, i.e., integrating- and non-integrating approaches, encountered major problems: low reprogramming efficiency, slow kinetics, and complicated procedure. For the integrating approaches of retrovirus and lentivirus-mediated four individual TFs delivery, the reprogramming efficiency was only ∼0.01% [30], [31]. Carey et al. generated human iPS cells from neonatal human keratinocytes, which are a potential cell source to be reprogrammed with high reprogramming efficiency (∼1%) [32], by using a single 4F2A polycistronic lentivirus vector thereby conferring a much lower reprogramming efficiency (∼0.00001%) [22]. Similar to our initial trials, we failed to generate human iPS cells by using our *OKSM*-transgene reprogramming system alone despite documented successful higher viral transduction. A plausible reason for the lower efficiency is that the stoichiometry of factor expression from the polycistronic vector is due to the 2A polycistronic vector system support near equimolar protein expression in vivo [21], which may be suboptimal for inducing reprogramming [22], [33].

Recently, several chemicals were reported to either enhance reprogramming efficiencies or substitute for specific reprogramming factors, such as BIX, VPA, butyrate, SB431542, PD0325901, A83-01, and PS48, etc [9], [16], [17], [19], [34], [35]. Among these small molecules, butyrate, a histone deacetylase inhibitor, relaxes the chromatin structure and significantly enhances reprogramming efficiency [16], [17]. SB431542, an inhibitor of TGFβ receptor, and PD0325901, an inhibitor of MEK-ERK signaling pathway, improve the reprogramming efficiency of reprogrammed somatic cells infected withwith TFs [18]. Notably, combination of A-83-01 and PD0325901 generated iPS cells from neonatal human epidermal keratinocytes by only two TFs [19]. For the first time, we used our *OKSM* fusion transgene system and demonstrated that a three compound combination (butyrate+SB431542+PD0325901) dramatically improved the reprogramming efficiency (∼0.93%), compared to either single butyrate treatment (∼0.29%) or previous reported two compound combination (SB431542+PD0325901) (∼0.05%).

Our reprogramming approach, which consists of a polycistronic vector plus a small molecule cocktail have several important implications. First, generation of hiPS cells by had a high percentage of full-reprogrammed iPS cells, facilitating the efficient isolation of desired colonies. Second, although our *OKSM* polycistronic vector alone did not successfully generate human iPS cells, our vector system provides a potential platform for high-throughput screening of novel small molecules that can enhance reprogramming process and significantly improve generation of human iPS cells. More importantly, identifying these potential candidate small molecules will facilitate discerning the reprogramming mechanisms. Third, future studies should test whether delivering the *hOKSM* fusion gene into human somatic cells via episomal vectors together with different small molecule cocktail could efficiently reprogram human somatic cells into human iPS cells that are not just transgene-free, but also have less or no mutations in their genome [36], [37], [38]. Generation of such transgene- and mutation-free iPS cells is critical for future clinical application.

## Materials and Methods

### Ethics Statement

All mouse work was conducted according to guidelines approved by the Maine Medical Center Research Institute Animal Care and Use Committee. This study was performed after approval by the Maine Medical Center Research Institute Review Board for Ethics and Safety (approval ID # 3674X).

### Plasmid construction

*Oct4*, *Klf4*, *Sox2*, and *c-Myc* were amplified by polymerase chain reaction (PCR) using Phusion™ High-Fidelity DNA Ploymerase (New England Biolabs), and then in-frame linked by E2A, T2A, and P2A sequence [21], respectively, as a single open reading frame (ORF) designated as *OKSM*. The *OKSM* fusion gene was then cloned in pMIGR1 retroviral vector, and the resulting plasmid was designated as pMIGR1-*hOKSM*. Retroviral expression vectors expressing each hTF were generated by PCR and conventional cloning methods, and designated as pMIGR1-*hOct4*, pMIGR1-*hKlf4*, pMIGR1-*hSox2*, and pMIGR1-*hMyc*.

### Transfection and western blot analysis

pMIGR1-*hOKSM* was transfected into U2OS cells in a 24-well plate by Fugene HD (Roche). Forty-eight hours after transfection, the cells were washed with cold PBS and lysed with RIPA lysis buffer plus protease inhibitor cocktail (Sigma). The cell lysates were separated by electrophoresis on 12% SDS-polyacrylamide gel and transferred to a nitrocellulose membrane (Pierce). The blot was blocked with TBST (20 mM Tris-HCl, pH 7.6, 136 mM NaCl, and 0.05% Tween-20) containing 5% non-fat milk, and then incubated with primary antibody solution at 4°C overnight. After washing with TBST, the membrane was incubated with horseradish peroxidase-conjugated secondary antibody for one hour at room temperature. The signals were detected with the Immobilon Western Chemilumminescent horseradish peroxidase substrate (Pierce). The list of primary antibodies is included in the Supporting Information (Table S3).

### Cell lines and cell culture

The HFF-1 and human hepatocytes were purchased from Millipore. The primary foreskin fibroblasts' medium was purchased from Millipore. For HFF-1 and human hepatocytes, the cells were cultured in DMEM containing 10% fetal bovine serum (FBS), 1× non-essential amino acid, 2 mM L-glutamine, and 1% penicillin-streptomycin.

### Retrovirus production

The 293T cells were plated at 8×10^5^ cells per 60-mm dish, cultured overnight, and then transfected with a mixture of DNA containing 2.5 µg of pMIGR1-*hOKSM* or pMIGR1 expression vectors expressing an individual TF (or pMIGR1-*hOct4*, pMIGR1-*hKlf4*, pMIGR1-*hSox2*, and pMIGR1-*hMyc*) and 1.5 µg of pCL-Ampho (IMGENEX) by Fugene HD (Roche), according to the manufacturer's instruction. The supernatant of the transfected cells was collected 24 hours after transfection and filtered through a 0.45 µm pore-size filter.

### iPS cell generation

Fibroblasts were seeded in a 12-well plate at 1×10^4^ cells per well at one day before transduction, and incubated with the virus-containing supernatant supplemented with 4 µg/ml polybrene (Sigma), followed by centrifugation (900 g for 30 min). Four days post-infection, the cells were split by using 0.025% trypsin-EDTA and plated on MEF feeder cultured in fibroblast medium. After 24 hours, the medium was switched to the human reprogramming medium (DMEM/F12, 20% knockout serum replacement, 1× non-essential amino acid, 2 mM L-glutamine, 0.1 mM 2-mercaptoethanol, 20 ng/ml FGF-2 and 1% penicillin-streptomycin), and treated with 0.5 mM sodium butyrate (Sigma), 2 µM SB431542 (Stemgent), and 0.5 µM PD0325901 (Stemgent) or other combinations of the compounds. The media were changed every other day until the induced colonies were picked up based on human ES cell colony morphology at days 20–23 post-infection. The GFP ratio was determined by flow cytometry at day-4 after viral infection. The individual ES cell-like colony was monitored for GFP marker expression during reprogramming.

### Immunofluorescent staining

The cells were fixed in 4% paraformaldehyde for 20 min at room temperature, washed three times with PBS, and blocked for 30 min with 5% FBS in PBS containing 0.05% Triton X-100, followed by incubation with primary and second antibodies. The antibodies were diluted in 1% FBS in PBS containing 0.05% Triton X-100. The list of primary antibodies is included in the Supporting Information (Table S3).

### Extraction of genomic DNA and RNA and RT-PCR

The genomic DNA was extracted by using QIAamp DNA Mini kit (Qiagen). All of the RNA was extracted by using Quick-RNA™ MicroPrep kit (Zymo Research). All of the RNA (200 ng) was used to synthesize cDNA using SuperScript III First-Strand Sunthesis SuperMix kit (Invitrogen), according to the manufacturer's instructions. PCR was performed with gene-specific primers. The list of primers is included in the Supporting Information (Table S2 and S3).

### Determination of retroviral integration sites by splinkerette-PCR

The retroviral integration sites in the hiPS cell lines were determined by splinkerette-PCR according to the previously reported method [29]. Briefly, genomic DNA (2 µg) was isolated from hiPS cells using Quick-gDNA MiniPrep Kit (ZYMO RESEARCH), and completely digested with Sau3A I restriction enzyme at 37°C overnight. The fragments were ligated with splinkerette-adaptor mixture by T4 DNA ligase (New England Biolabs) at 4°C overnight. The adaptor-ligated splinkerette products were digested with Mse I for 6 hours at 37°C and then purified by QIAquick Gel Extraction Kit (QIAGEN) according to the manufacturer's instructions. The purified products were processed for primary PCR amplification by the primary primers Splink-F1 and 5′LTR-R1. A secondary PCR was then carried out on 1 µl of the primary PCR product with the primers Splink-F2 and 5′-LTR-R2. The resulting PCR products were run on a 4% agarose gel. The sequences for each primer are included in the Supporting Information (Table S2 and S3).

### Karyotype analysis

The cells were cultured for 24 hours in a CO_2_ incubator, and treated with colcemid acid (0.1 µg/ml, final concentration) for one hour before harvesting. The cells were washed with PBS, transferred into 15 ml tubes, and then centrifuged for 10 min at 250 g. The supernatant was removed and resuspended in 10 ml KCl (75 mM). The cell mixtures were incubated for 30 min in 37°C water bath and then fixed by adding 2 ml of fixative solution (methanol/acetic acid 3∶1). The fixed cells were washed at least twice with 10 ml of fixative solution before being applied onto chilled slides. The slides with chromosomes were dried and treated with 0.025% trypsin for 5 min and stained with KaryoMAX Giemsa Stain solution (Invitrogen) for 5 min.

### Calculation of reprogramming efficiency

We quantified infected cells (GFP^+^) at day-4 post-infection and counted the ES cell-like cell colonies, AP^+^ colonies, and Tra-1-60^+^ colonies after reprogramming. Reprogramming efficiency was determined by the total number of true pluripotent iPS cell colonies divided by the total number of infected cells (GFP^+^).

### *In vitro* differentiation of iPS cells

For EB formation, human iPS cells were expanded and harvested by treatment with Collagenase IV (STEMCELL). The clumps of the cells were transferred to ultra-low attachment plate in DMEM/F12 containing 20% knockout serum replacement, 1× non-essential amino acid, 2 mM L-glutamine, 0.1 mM 2-mercaptoethanol and 1% penicillin-streptomycin. The medium was changed every other day. After eight days, the EBs were placed in pre-condition culture for 2–3 days and maintained in three different differentiation medium in ultra-low attachment plates. For endoderm differentiation, the EBs were cultured in the presence of KO-DMEM medium supplemented with 10% FBS, 2 mM L-gultamine, 0.1 mM 2-mercaptoethanol, 1× non-essential amino acids, and 1% penicillin-streptomycin. For mesoderm differentiation, we used the same medium described above, and added ascorbic acid (0.5 mM). For ectoderm induction, the EBs were cultured in N2/B27 medium (Invitrogen). After the precondition step, the EBs were transferred to a 0.1% gelatin-coated plate and cultured in differentiation medium for two weeks. The medium for each condition was changed every other day.

### Teratoma formation assay

All mouse procedures were conducted in compliance with institutional animal use guidelines. The human iPS cells were collected from MEF feeder by Accutase (Millipore), washed three times with cold PBS, and then resuspended in PBS supplemented with 50% Matrigel (BD Biosciences). The cell mixtures were kept on ice and drawn into 1-ml syringe immediately before injection. Approximately 5×10^6^ cells in 200 µl per injection site were used. NOD/SCID mice were injected in the dorsolateral area into the subcutaneous space on both sides. The tumors were dissected from mice 8–10 weeks after injection, fixed in 4% formaldehyde, and then embedded in paraffin. The paraffin section were processed and stained with hematoxylin and eosin.

## Supporting Information

Table S1 **Estimation of reprogramming efficiency.**
(DOC)

Table S2 **List of primers for qPCR.**
(DOC)

Table S3 **List of antibodies for immunofluorescence staining and western blot.**
(DOC)
