# Correction: Efficient Generation of Fully Reprogrammed Human iPS Cells via Polycistronic Retroviral Vector and a New Cocktail of Chemical Compounds

**DOI:** 10.1371/annotation/2a5e8c51-2ad9-47c7-a4dd-9c9616bab32a

**Published:** 2012-09-05

**Authors:** Zhonghui Zhang, Yongxing Gao, Albert Gordon, Zack Z. Wang, Zhijian Qian, Wen-Shu Wu

The following information was missing from the Funding section: This work was also partially supported by the Bioinformatic and Genomics Core Facility at Maine Medical Center Research Institute supported by 5P20RR18789 (PI: Don Wojchowski) from the National Center for Research Resources at the NIH.

