# Supplementary material for: Efficient Generation of Fully Reprogrammed Human iPS Cells via Polycistronic Retroviral Vector and a New Cocktail of Chemical Compounds
Source: PLoS One. 2011 Oct 26;6(10):e26592. doi: 10.1371/journal.pone.0026592 (PMC3202534; doi:10.1371/journal.pone.0026592)
Supplement: Table S1 — Estimation of reprogramming efficiency. (DOC) [file pone.0026592.s001.doc]

**Table S1: Estimation of reprogramming efficiency**

| Experiment | Source of cells | Treatment | GFP+ cells | Number of human ES cell-like colonies | Efficiency |
| --- | --- | --- | --- | --- | --- |
| 1 | human hepatocytes | Without NaB | 1.8× 10^4^ | 0 | 0 |
| 2 | human hepatocytes | Without NaB | 1.8× 10^4^ | 0 | 0 |
| 3 | human hepatocytes | NaB | 1.8× 10^4^ | 134 | 0.76% |
| 4 | human hepatocytes | NaB | 1.8× 10^4^ | 115 | 0.65% |
| 5 | HFF-1 | Without NaB | 1.0× 10^4^ | 0 | 0 |
| 6 | HFF-1 | Without NaB | 1.0× 10^4^ | 0 | 0 |
| 7 | HFF-1 | NaB | 1.0× 10^4^ | 252 | 2.38% |
| 8 | HFF-1 | NaB | 1.0× 10^4^ | 204 | 1.94% |
