# Supplementary material for: Efficient Generation of Fully Reprogrammed Human iPS Cells via Polycistronic Retroviral Vector and a New Cocktail of Chemical Compounds
Source: PLoS One. 2011 Oct 26;6(10):e26592. doi: 10.1371/journal.pone.0026592 (PMC3202534; doi:10.1371/journal.pone.0026592)
Supplement: Table S2 — List of primers for qPCR. (DOC) [file pone.0026592.s002.doc]

**Table S2: List of primers for qPCR and PCR**

| **Primer** | **Sequence (5’ to 3’)** | **Application** |
| --- | --- | --- |
| Oct4-rtF | ACAACAATGAAAATCTTCAGGAGATATGC | qPCR |
| Oct4-rtR | ACAGAACCACACTCGGACCACATCCTTC | qPCR |
| Sox2-rtF | GCTCCATGGGTTCGGTGGTCAAGTCCGAG | qPCR |
| Sox2-rtR | CGCTCTGGTAGTGCTGGGACATGTGAAGTC | qPCR |
| Nanog-rtF | CATCCTGAACCTCAGCTACAAACAGGTGAAG | qPCR |
| Nanog-rtR | GGGTAGGTAGGTGCTGAGGGCTTCTGCG | qPCR |
| REX1-rtF | AGAGACATTTCCTGGTTCATACTGGAGAG | qPCR |
| REX1-rtR | CTTGTTCGTATTTGCATGCGTTAGGATGTG | qPCR |
| GDF3-rtF | CAGAAGTTCCAACCTGTGCCTTATATCTTG | qPCR |
| GDF3-rtR | TCTTTGGGTAAAGAAAGAAACCTTGGTC | qPCR |
| DAPP5-rtF | GACGCGGCTGCTGAAAGCCATTTTCGGCC | qPCR |
| DAPP5-rtR | CTTCGGCAAGTTTGAGCATCCCTCGCTC | qPCR |
| UTF1-rtF | CGCCGGCTCCCAGCGAACCAGACGCCAC | qPCR |
| UTF1-rtR | CGCGGACGGGCGCGCGGTCCTCG | qPCR |
| Lin28-rtF | CGTGTCCAACCAGCAGTTTGCAGGTGGC | qPCR |
| Lin28-rtR | AACCCTTCCATGTGCAGCTTACTCTGGTG | qPCR |
| GAPDH-rtF | ATTGACCTCAACTACATGGTTTACATG | qPCR |
| GAPDH-rtR | TTGGAGGGATCTCGCTCCTGGAAG | qPCR |
| Transgene-rtF | AGACTTCACATGTCCCAGCACTACCAGAG | PCR |
| Transgene-rtR | CATAGTTCCTGTTGGTGAAGCTAACGTTGAG | PCR |
| Splink-F1 | CGAAGAGTAACCGTTGCTAGGAGAGA | PCR |
| Splink-F2 | GTGGCTGAATGAGACTGGTGTCGAC | PCR |
| 5'LTR-R1 | TTAAGCTAGCTTGCCAAACCTAC | PCR |
| 5'LTR-R2 | TTAAGCTAGCTTGCCAAACCTACAGG | PCR |
