# Supplementary material for: Efficient Generation of Fully Reprogrammed Human iPS Cells via Polycistronic Retroviral Vector and a New Cocktail of Chemical Compounds
Source: PLoS One. 2011 Oct 26;6(10):e26592. doi: 10.1371/journal.pone.0026592 (PMC3202534; doi:10.1371/journal.pone.0026592)
Supplement: Table S3 — List of antibodies for immunofluorescence staining and western blot. (DOC) [file pone.0026592.s003.doc]

**Table S3: List of antibodies:**

| **Protein** | **Supplier** | **Catalog number** | **Use** | **Dilution** |
| --- | --- | --- | --- | --- |
| Oct4 | Cell Signaling | #2788 | WB | 1:1000 |
| Klf4 | Cell Signaling | #4038 | WB | 1:1000 |
| Sox2 | Cell Signaling | #2748 | WB | 1:1000 |
| c-Myc | Cell Signaling | #9402 | WB | 1:1000 |
| GAPDH | Bethyl | A300-641A | WB | 1:10000 |
| Tra-1-60 | Stemgent | 09-0068 | ICC | 1:100 |
| Tra-1-81 | Stemgent | 09-0069 | ICC | 1:100 |
| SSEA4 | Stemgent | 09-0006 | ICC | 1:100 |
| Oct4 | Stemgent | 09-0023 | ICC | 1:100 |
| Sox2 | Stemgent | 09-0024 | ICC | 1:100 |
| Tuj1 | R&D Systems | MAB1195 | IHC, WB | 1:200 |
| Anti-Actin, smooth muscle (ASM) | Millipore | CBL171 | IHC, WB | 1:200 |
| AFP | R&D Systems | MAB1368 | IHC, WB | 1:200 |
|  |  |  |  |  |
|  |  |  |  |  |
|  |  |  |  |  |
|  |  |  |  |  |
